# Adaptive responses in a PARP inhibitor window of opportunity trial illustrate limited functional interlesional heterogeneity and potential combination therapy options

**DOI:** 10.18632/oncotarget.26947

**Published:** 2019-05-28

**Authors:** Marilyne Labrie, Tae-Beom Kim, Zhenlin Ju, Sanghoon Lee, Wei Zhao, Yong Fang, Yiling Lu, Ken Chen, Pedro Ramirez, Michael Frumovitz, Larissa Meyer, Nicole D. Fleming, Anil K. Sood, Robert L. Coleman, Gordon B. Mills, Shannon N. Westin

**Affiliations:** ^1^ Knight Cancer Institute and Cell, Developmental and Cancer Biology, Oregon Health and Science University, Portland, OR, USA; ^2^ Department of Bioinformatics and Computational Biology, University of Texas, MD Anderson Cancer Center, Houston, TX, USA; ^3^ Department of Systems Biology, University of Texas, MD Anderson Cancer Center, Houston, TX, USA; ^4^ Department of Gynecologic Oncology and Reproductive Medicine, University of Texas, MD Anderson Cancer Center, Houston, TX, USA

**Keywords:** poly (ADP-ribose) polymerase inhibitor, combination therapy, adaptive response, ovarian cancer, targeted therapy

## Abstract

Poly (ADP-ribose) polymerase inhibitor (PARPi)-based combination therapies are demonstrating efficacy in patients, however, identifying the right combination for the right patient remains a critical challenge. Thus, it is urgent to develop approaches able to identify patients likely to benefit from specific combination therapies. Several groups, including ours, have demonstrated that targeting adaptive responses induced by PARPi increases depth and duration of response. In this study, we instituted a talazoparib (PARPi) monotherapy window of opportunity trial to identify informative adaptive responses in high grade serous ovarian cancer patients (HGSOC). Patients were treated for 7 to 14 days with PARPi monotherapy prior to surgery with tissue samples from multiple sites being collected pre- and post-treatment in each patient. Analysis of these samples demonstrated that individual patients displayed different adaptive responses with limited interlesional heterogeneity. Ability of combination therapies designed to interdict adaptive responses to decrease viability was validated using model systems. Thus, assessment of adaptive responses to PARPi provides an opportunity for patient-specific selection of combination therapies designed to interdict patient-specific adaptive responses to maximize patient benefit.

## INTRODUCTION

High grade serous ovarian cancer (HGSOC) is associated with a five-year overall survival (OS) rate of 47% and is characterized by *TP53* mutations and chromosomal instability [1, 2]. Importantly, defects in the homologous recombination (HR) DNA repair can be detected in roughly 50% of HGSOC [3, 4]. Homologous recombination deficiency (HRD) results in a therapeutic liability that leads to synthetic lethality with poly (ADP-ribose) polymerase inhibitors (PARPi) [5, 6].

PARPi monotherapy has demonstrated activity in solid tumors with optimal activity in tumors with BRCA1/2 mutations or HRD [7–12]. As a result, multiple PARPi (olaparib, talazoparib, rucaparib and niraparib) have been approved or are pending approval by the FDA for ovarian, breast, pancreas and prostate cancers [13–16]. While PARPi monotherapy, particularly in HRD tumors, markedly improves progression free survival (PFS), the effects on OS have been more limited [17]. Rapid development of resistance to PARPi monotherapy likely contributes to the limited effects on overall survival [18]. Several PARPi resistance mechanisms have been reported including, acquisition of mutations that restore the reading frame of a mutated gene such as *BRCA1* or *Rad51* [19, 20], increased drug efflux [21, 22], increased HR activity through 53BP1 downregulation [23] and loss or mutation of PARPi [24]. To counteract these mechanisms, several groups, including our own, have suggested the use of PARPi-based combination therapies [15, 18, 25–29]. As a result, multiple clinical trials are underway to test whether combination therapy increases the depth and duration of response, expands the spectrum of patients who benefit from PARPi or resensitizes PARPi resistant tumors to PARPi. These trials include combination of PARPi with conventional chemotherapy (NCT03259503), PD-L1 (NCT02734004; NCT02657889), WEE1 (NCT02511795), ATR (NCT03462342), PI3K (NCT02511795), Akt/mTOR (NCT02208375) and MEK (NCT03162627) inhibitors [30]. However, a key challenge in these studies is to choose the right drug combination for the right patient, as each tumor has the potential to engage a different set of resistance mechanisms and thus only benefit from specific combinations.

Adaptive responses to targeted therapies can allow cancer cells to survive therapeutic stress until they develop genomic or epigenomic acquired resistance [31]. Adaptive responses, which can occur early in therapy, are best identified by assessment of changes in protein levels and in particular post-translational modifications associated with functional activation. Thus, early implementation of combination therapy to interdict adaptive responses could avoid development of acquired resistance. In cell line models, adaptive response to PARPi can be detected after a few hours of treatment and, importantly, individual cell lines display distinct adaptive responses [27, 32]. We hypothesized that we would detect patient-specific adaptive responses to PARPi early during the course of treatment that could predict combination therapies for individual patients. Further, we hypothesized that there would be limited interlesional heterogeneity in adaptive responses to PARPi further supporting the utility of the approach. Window of opportunity trials have provided valuable information about new therapies without compromising patient outcomes [33]. Thus, we tested these concepts through a window of opportunity study wherein HGSOC patients were treated with monotherapy talazoparib between the diagnosis of their disease and cytoreductive surgery. Our results indicate that adaptive responses can be identified early during the course of PARPi treatment with limited interlesional heterogeneity and that individual patients display different adaptive responses to talazoparib, reinforcing the need for selection of combination therapies specific for each patient. This further raises the potential that assessing adaptive responses to PARPi will allow selection of patient-specific combination therapies that will maximize benefit.

## RESULTS

Four patients with HGSOC were enrolled into this study between July and December 2015. Clinical and demographic characteristics are described in Table 1. Related adverse events experienced by the patients during the window period were all grade 1 and included anorexia, abdominal distention, constipation, fatigue, nausea, pain, and urinary frequency (*n* = 1 for each symptom).

**Table 1:** Demographic and clinical characteristics of patients treated with talazoparib induction therapy

Patient	Age	Race	Stage	Histology	BRCA status	Days of Talazoparib
1	72	White	IIIc	High grade serous	WT	13
2	55	White	IIIc	High grade serous	BRCA1^*^	7
3	61	Black	IIIc	High grade serous	WT	10
4	58	White	II	High grade serous	BRCA1	14

^*^This BRCA1 mutation was considered as non-functional.

**Figure 1:**
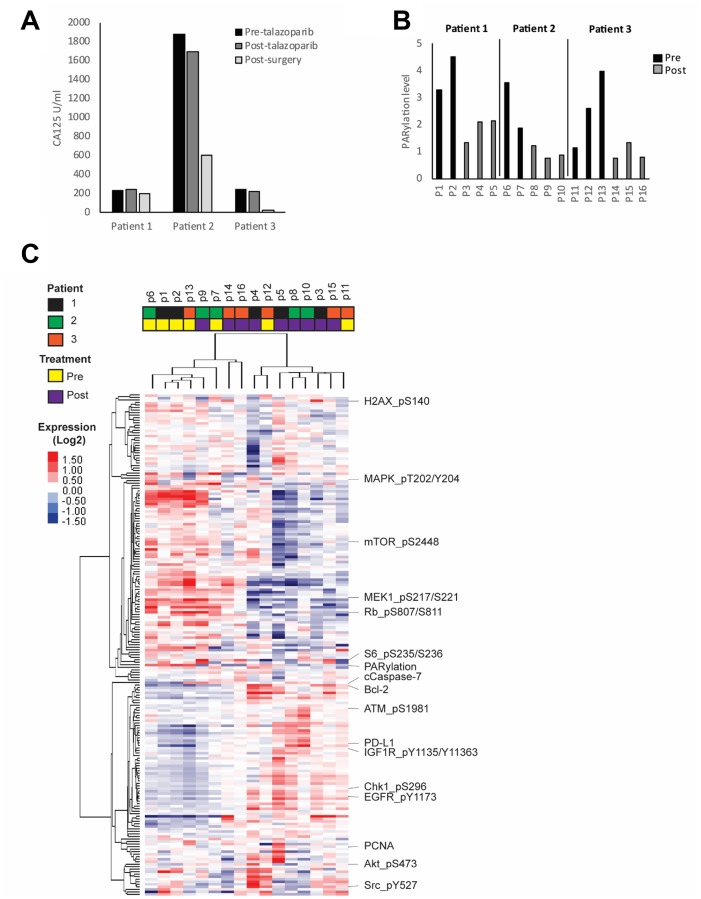
HGSOC response to PARP inhibitor. Patients were treated for 7 to 14 days with talazoparib. Blood and tumor samples were collected before and after treatment. (**A**) Plasma CA125 concentration was measured before and after treatment as well as after tumor reductive surgery. (**B**) PARP enzymatic activity was assessed in all pre- and post-treatment tumor samples by measuring the level of PARylation using RPPA analysis. (**C**) Heat map representing the unsupervised clustering of protein samples analyzed by RPPA. Red and blue colors represent higher and lower expression, respectively.

Three patients had viable tumor from multiple sites pre- and post-treatment with talazoparib available for analysis. DNA sequencing of the tumors reveled that all three patient tumors displayed *TP53* mutations. Moreover, patient 1 had a mutation in *TBX3*, patient 2 displayed mutations in *KDM6A*, *PIK3CG*, *TOP2A*, *MLL* and *SMC3*, and patient 3 had a mutation in *NOTCH1*. Patient 1 demonstrated a 4% increase in blood levels of the CA125 biomarker during the course of treatment. Conversely, patient 2 and 3 both demonstrated a 10% decrease in CA125 (Figure 1A).

To investigate heterogeneity of adaptive responses to talazoparib, we collected multiple pre- and post-treatment tumor samples from different sites in the peritoneal cavity from each patient (Supplementary Table 1). To assess changes in protein and phosphoprotein levels in the small samples available we analyzed expression of a panel of 300 proteins emphasizing post-translationally modified proteins using RPPA that we have extensively validated for this purpose [34–40]. Importantly, we detected PARPi target engagement in all post-treatment lesions as shown by markedly decreased PARylation levels as assessed by measuring PAR (total poly ADP ribosylated proteins) (Figure 1B).

We next investigated inter-lesion heterogeneity and drug response in each patient through unsupervised clustering of the protein expression data. We found that samples tended to cluster according to treatment status with clear changes in most post-treatment samples when compared to pre-treatment (Figure 1C, Supplementary Figure 1). While a number of the lesions from individual patients clustered together, there was significant heterogeneity across lesions from individual patients. There are multiple potential technical and biological reasons for the cluster patterns including inter-tumoral heterogeneity in terms of tumor and stromal content or differences in intra-tumoral drug concentrations.

**Figure 2:**
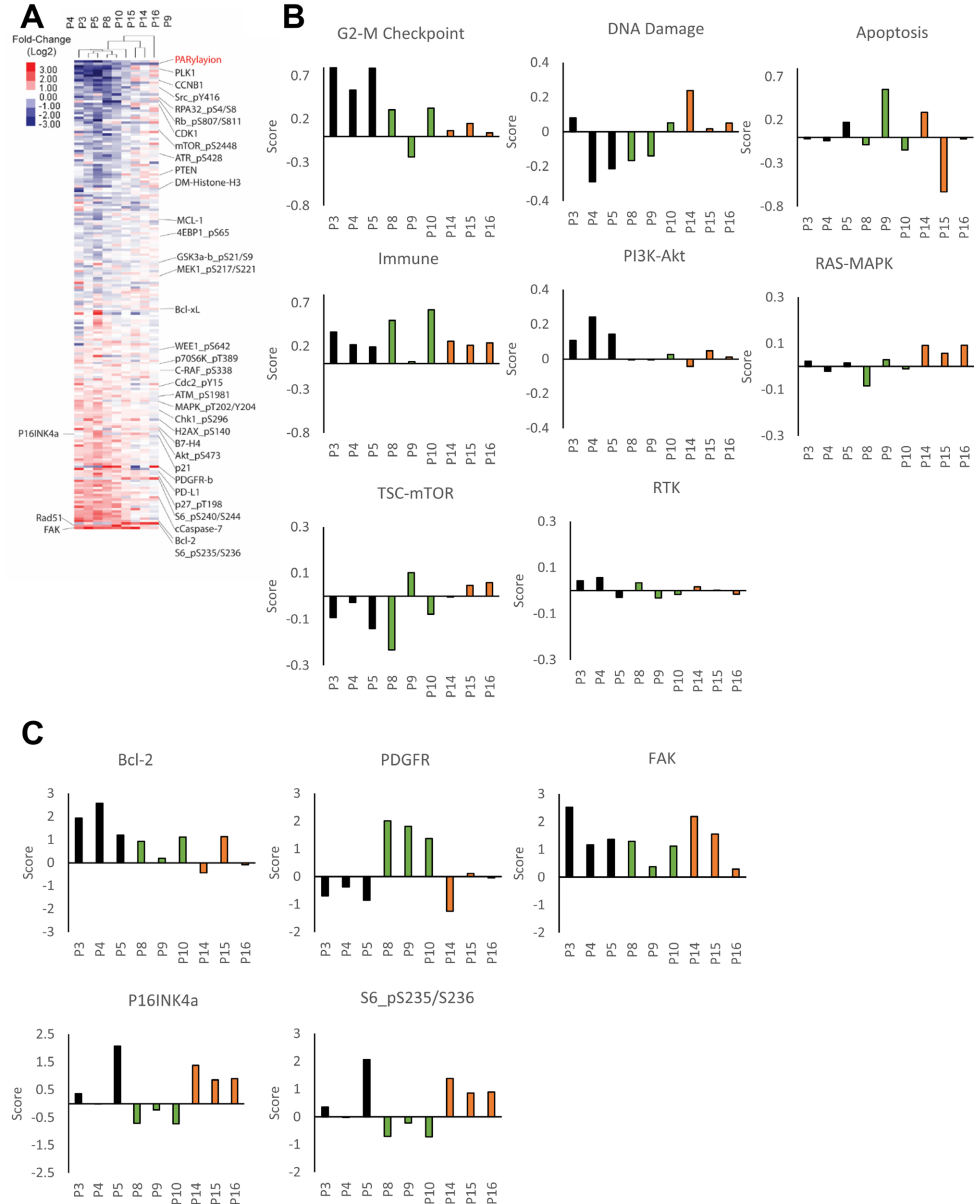
Patient-specific adaptive response to PARP inhibitor. (**A**) The heat map represents unsupervised clustering of post-treatment samples normalized with the average of pre-treatment samples from that patient. Proteins were rank ordered according to the ratio of expression across patients. Samples P3-P5 are from patient 1, P8-P10 from patient 2 and P14-P16 from patient 3. (**B**) Pathway activity was assessed using pathway scores. The histogram represents the change of each post-treatment sample compared to the average of pre-treatment samples. (**C**) Histograms representing the change of protein expression (Z-score) in each post-treatment samples compared to the average of pre-treatment samples. Samples P3-P5 are from patient 1, P8-P10 from patient 2 and P14-P16 from patient 3.

To analyze the adaptive response of individual patient tumors to PARPi and mitigate patient-specific characteristics in each tumor, we normalized protein expression of each post-treatment sample by the average of all pre-treatment samples from that patient. We next applied unsupervised clustering of patient samples against the ranked sum of protein expression from the most downregulated to the most upregulated proteins (Figure 2A). This analysis showed that the samples clustered by patients, with the exception of sample P9 from patient 2. Sample 9 did demonstrate PARP engagement as indicated by decreased PARylation (Figure 1B) as well as by a marked increase in PDGFR (Figure 2A). Overall, there was a conserved pattern of downregulated and upregulated proteins across patient 1 and patient 2 (with the exception of sample 9), suggesting a degree of commonality in response to PARPi.

To explore responses in different patients and across lesions, we used pathway score analysis to determine which major pathways were altered by PARPi in each patient (Figure 2B, Supplementary Table 3) as well as analysis of specific proteins (Figure 2C, Supplementary Table 3). Pathway scores were determined based on basal expression level and changes in the expression of proteins known to be involved in activity of the pathway based on approaches described previously (Supplementary Table 2) [41]. All of the lesions in patient 1 demonstrated an increase in G-2M checkpoint, immune and PI3K-AKT pathway activation as well as selective increases in BCL2 and FAK. Two of the three lesions in patient 2 (with the exception of sample 9 indicated above) showed increases in the G2-M checkpoint and immune pathways, FAK and a modest increase in BCL2 (Figure 2B, 2C). There was a remarkable patient specific increase in the PDGFR receptor including sample 9 (Figure 2C). Patient 3 showed a remarkably different pattern of adaptive responses, with a modest G2-M checkpoint, DNA damage, immune and RAS-MAPK pathway activation as well as induction of FAK, p16 and phospho-S6.

**Figure 3:**
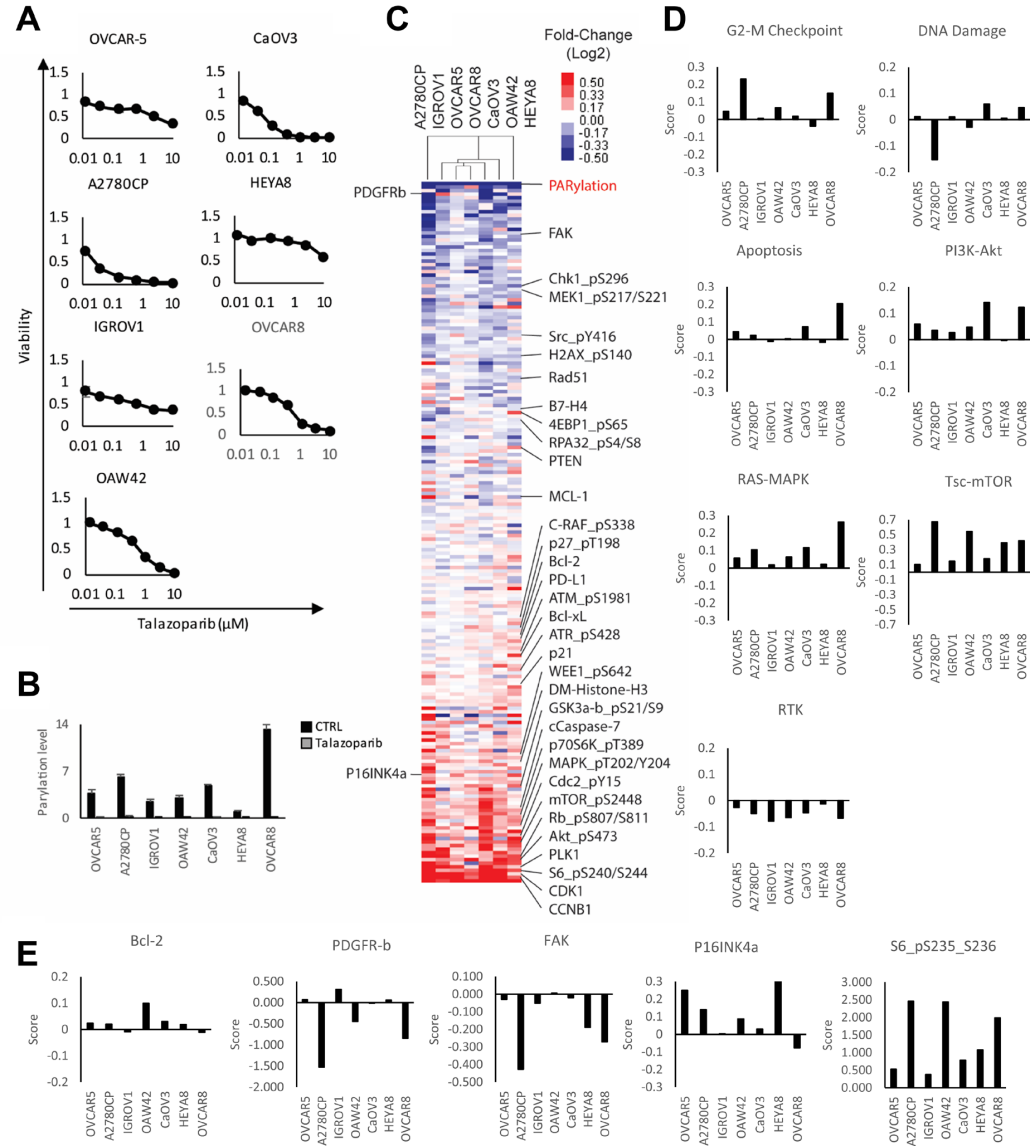
Adaptive response of ovarian cancer cell lines treated with PARP inhibitor. (**A**) Viability of cell lines treated with increasing concentration of talazoparib for 6 days. Viability was measured using Prestoblue assay. (**B**) IC50 was measured and cells were treated for 72 hours with or without IC50 doses of talazoparib determined experimentally for each line. PARP enzymatic activity was assessed by measuring the level of PARylation using RPPA analysis. (**C**) The heat map represents unsupervised clustering of post-treatment samples normalized with pre-treatment samples and rank sum ordering of the protein expression. The red and blue colors represent upregulated and downregulated proteins, respectively. (**D**) Pathway activity was assessed using pathway scores. The histogram represents the change of each treated sample compared to control. (**E**) Histograms representing the change of protein expression (Z-score) in each PARPi treated cell lines compared to untreated.

We have shown that combination therapy targeting adaptive responses to PARPi observed in model systems can result in synergism both *in vitro* and *in vivo* [27, 28, 42]. To determine whether the adaptive response observed in patients would also be observed in cell lines, providing models to explore the relevance of adaptive responses observed in samples from the window of opportunity trial, we treated a panel of seven gynecological cancer cell lines for six days with or without talazoparib. As shown in Figure 3A, each cell line had differential sensitivity to the drug, with A2780CP and CaOV3 being the most sensitive and HEYA8, OVCAR5 and IGROV1 the most resistant. OAW42 and OVCAR8 had an intermediate level of sensitivity. Notable, these differences were not dependent on mutation status of *BRCA1/2* since none of these cell lines display *BRCA1/2* alterations, with the exception of OVCAR8, that has been reported to exhibit loss of heterogeneity for both *BRCA1/2* and methylation of *BRCA2* promoter [43] but was resistant to PARPi. We treated cells for 3 days with the IC50 dose of talazoparib determined specifically for that line and then analyzed protein expression by RPPA. Similar to the patients, all cell lines displayed a marked inhibition of PARylation following PARPi treatment, indicative of adequate dosing and target engagement (Figure 3B). The overall protein changes observed in cell lines were consistent with those observed in patient samples (Figure 3C) and indeed was more consistent than that observed in the patients. This could reflect the failure of the cell lines to reflect the complex tumor microenvironment present in the patient samples. More importantly, similar to the patients, each cell line had a distinctive adaptive response as shown by pathway score analysis (Figure 3D, Supplementary Table 4) and increases in specific proteins (Figure 3E). For instance, while the G2/M checkpoint was increased in many cell lines, the response was most striking in A2780CP and OVCAR8. This heterogeneity was observed across the different pathways as well as proteins of interest with the exception of the PDGFRA induction observed in patient 2. This suggested that cell lines could be used to probe the functional consequences of PARPi in patient samples.

**Figure 4:**
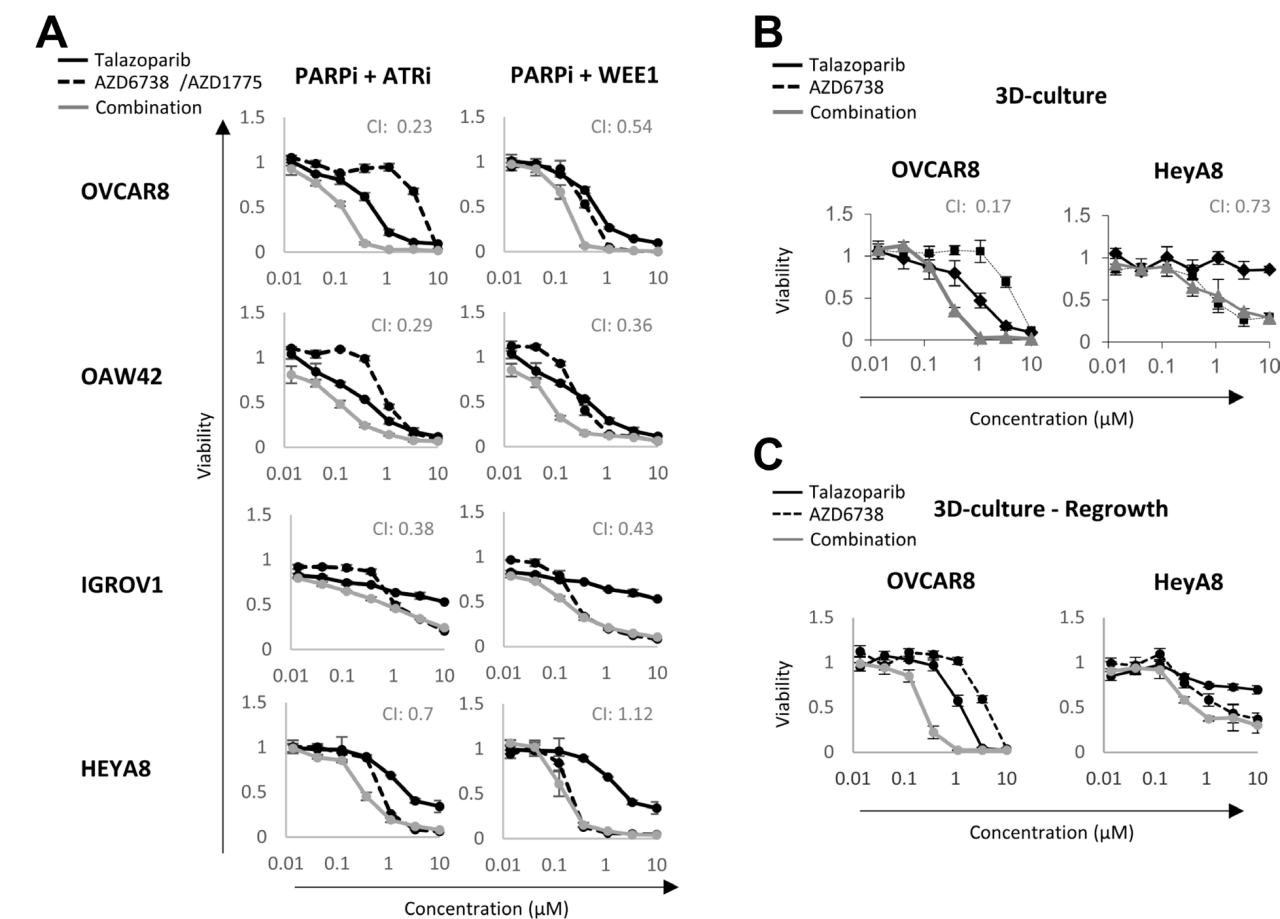
Synergism between PARP and DNA damage checkpoint inhibitors. (**A**) Cancer cell lines were treated with or without talazoparib, AZD6738/AZD1775 and their combination for 6 days. Viability was measured using Prestoblue assay. (**B**) 3D cell viability assay of OVCAR8 and HEYA8 treated with or without talazoparib, AZD6738 or their combination for 7 days. (**C**) After 7 days of treatment, drugs were washed out and cells were allowed to grow for an additional 7 days before a Prestoblue viability assay. CI: Chou-Talalay combination index. CI = 1 represents additivity, CI < 1 indicates synergism and CI > 1 indicates antagonism. A CI < 0.4 indicates a strong synergism.

We have previously demonstrated synergy between PARP and MEK inhibitors in lines with an activated RAS/MAPK pathway and for PARP and PI3K pathway inhibitors in cancer patients consistent with the concept that targeting adaptive responses could be effective [27, 44, 45]. Further, we have demonstrated that PARP and PD-L1 are synergistic as a consequence of induction of a STING response by PARP inhibitors [46, 47]. We thus decided to further test the potential for pathway analysis in the presence of talazoparib to predict response to drug combinations. In the presence of DNA damage, the G2/M checkpoint arrests cell cycle progression and allows time for DNA repair before mitosis [48]. Previous studies, including our own, have demonstrated synergism between G2/M DNA damage checkpoints inhibitors (WEE1, Chk1 or ATR) and PARPi in several cancer models [49–55]. We thus tested whether G2/M pathway scores would predict which cell lines would respond to combinations of PARPi with G2/M checkpoint inhibitors. We selected OVCAR8, OAW42, HEYA8 and IGROV1 for their medium to low single agent talazoparib sensitivity and performed viability assays in the presence or absence of talazoparib and WEE1 (AZD1775) or ATR (AZD6738) inhibitors (Figure 4A). Remarkably, the rank order of the G2/M pathway score (OVCAR8>OAW42>IGROV1>HEYA8 is mirrored in the CI index of the combination. Overall, the ATR inhibitor displayed a stronger synergism index in all cell lines when compared to the WEE1 inhibitor. A strong synergism index (CI < 0.5) was observed in all cell lines treated with PARP and ATR inhibitors, except for HEYA8 cells (RAS mutant) which had a modest synergism index (CI: 0.7). In PARP and WEE1 inhibitor treated cells, there was a strong synergism in OAW42 and IGROV1, a mild synergism in OVCAR8 and no synergism in HEYA8 cells. These results were consistent with the contention that pathway score predict the response of cell lines to the G2/M DNA damage checkpoint inhibitor combination and could potential predict benefit to the combination in patients.

We then compared the response of OVCAR8 and HEYA8 cell lines (highest and lowest G2/M pathway score) to talazoparib and AZD6738 in 3D-culture. The drug combination was strongly synergistic in OVCAR8 (CI: 0.17) but not in HEYA8 (CI: 0.73), which is consistent with HEYA8 displaying a lower G2/M checkpoint score in the presence of talazoparib (Figure 4B). A 3D regrowth experiment also confirmed the lethal effect of the drug combination in OVCAR8, as the cells had not recovered seven days after drugs were washed out (Figure 4C). Taken together, these results suggest that pathway scores provide an effective approach to identify and select targeting approaches for adaptive responses to PARPi. These may also prove useful in selecting combination therapy for patients.

## DISCUSSION

To our knowledge, this is the first window of opportunity study to be completed in patients with HGSOC. The goal of this study was to investigate heterogeneity of adaptive responses in multiple tissues of HGSOC patients treated with talazoparib for a short period of time. Our results indicate that: (1) adaptive responses to talazoparib can be detected after only 7 to 14 days of treatment, (2) each patient displays a distinct set of adaptive responses to PARPi that can be characterized through RPPA analysis and pathway scores, (4) as compared to marked heterogeneity in basal and treated patients samples, there was much less heterogeneity in adaptive responses with 8 or 9 samples showing similar responses within patients, (5) cell line models display similar adaptive response to talazoparib when compared to tumor samples, and (6) adaptive response analysis can be used as a predictive tool for response to combination therapies at least in model systems. Importantly, we have demonstrated the feasibility of window of opportunity studies in HGSOC as a novel strategy to improve our understanding of drug development. They also provide an opportunity to select combination therapies that may be effective in individual patients.

Targeted therapies offer an opportunity to change the course of ovarian cancer treatment; however, efficacy has been modest with the exception of PARPi in patients with abnormalities in *BRCA1/2*. In the case of PARPi, the first challenge is identification of patients that are more likely to respond to the drug and, importantly, might have a durable response [56]. Indeed, not all patients with known *BRCA* mutations respond to PARPi [13, 30, 56–58], which indicates the presence of underlying mechanisms of intrinsic and acquired drug resistance. Furthermore, many different mechanisms can lead to HR deficiency and a subset of HGSOC patients without apparent HRD appear to benefit from PARPi. Patients with HR defects represent up to 50% HGSOC patients with about 30% having alterations in *BRCA1/2* [4, 59]. These patients are more likely to respond to PARPi, but current genetic testing is insufficient to identify the complete population of patients who benefit from PARPi [56]. Further, combination therapy may be active in patients without defects in BRCA1/2 or HRD as we have demonstrated with PARP and PI3K pathway inhibitors [45]. The fact that we could detect responses to PARPi in as little as a week of treatment in patients with normal BRCA1/2 offers the opportunity to improve the decision-making process in regard to PARPi and PARPi combinations. This short window may also help identify tumors that display inherent resistance to the drug and suggest a rapid change in treatment to a more appropriate one. This could potentially decrease costs and toxicity and result in a patient receiving a more effective therapy earlier in their course of treatment. Further studies will be required to determine the optimum period of treatment to identify targetable adaptive responses.

The second challenge encountered with PARPi therapy is the development of drug resistance. Indeed, although multiple PARPi used as monotherapy have shown improved PFS, there has been little to no impact on OS, due in part, to the rapid development of drug resistance [18, 58]. This suggests that PARPi-based combination therapy may be required to interdict or overcome resistance [30, 32, 60]. The main problem with this strategy is determining which patients will respond to a particular combination. Indeed, in our reported studies with PARPi and PI3K inhibitors there were a subset of patients without BRCA1/2 abnormalities who demonstrated prolonged responses [45]. Similarly, in the MEDIOLA trial, which combines PARP and anti-PD-L1, there is a subset of patients with marked responses who cannot be adequately identified by PD-L1 levels alone [61]. An ability to identify patients likely to benefit from particular combinations in advance would greatly increase the utility of these combinations. Although further studies will be needed to test and improve our model, our results strongly suggest analysis of the adaptive response of cancer cells after a short period of treatment has potential to inform rational combination therapy with PARPi. Indeed, our results demonstrated that each patient had a distinct adaptive response to PARPi, which might be explained by the unique genotype of their tumors. A study including more patients would be necessary to determine if specific adaptive responses can be identified in groups of patients and associated to specific genetic alterations. More importantly, in all but one case, tumors from the same patient that were spatially separated displayed similar adaptive responses. HGSOC rapidly spreads inside the peritoneal cavity and tumors with different spatial locations have been reported to be highly heterogeneous, rendering treatment more difficult [62–64]. Our results indicate that underlying molecular features can drive drug response across different sites and might be targeted through personalized combination therapies. Interestingly, harvesting tumor samples from distinct sites and using proteomics analysis provides a new approach to assess inter-tumoral heterogeneity at a functional level.

The adaptive responses seen in each patient suggest opportunities for combination therapies. For example, the consistent activation of pathways and targets across all lesions including G-2M checkpoint, immune and PI3K-AKT pathway activation and increases in BCL2 and FAK suggest a number of potential combinations including DNA damage checkpoint inhibitors, anti-PD-L1 or PD1 monoclonal antibodies, PI3K-AKT pathway inhibitors, or BCL2 or FAK inhibitors. Indeed, a clinical trial of PARP and PI3K inhibition demonstrated activity in HGSOC [45]. The consistent responses with a modest G2-M checkpoint, DNA damage, immune and RAS-MAPK pathway activation as well as induction of p16 and phospho S6 across all three lesions suggests combination with DNA damage checkpoint inhibitors, anti-PD-L1 or PD1 monoclonal antibodies, or RAS-MAPK or mTOR pathway inhibitors. The heterogeneity in responses in patient 2 raises a more challenging question of whether interdiction of adaptive responses found in 2 of 3 lesions would result in patient benefit. However, even in patient 2 the marked induction of the PDGFR receptor in all 3 lesions provides a potential therapeutic opportunity. In terms of a note of caution, the induction of multiple adaptive resistance pathways in each patient raises the potential concern that blocking a single adaptive response may not be effective. Whether combinations with more than 2 drugs such as the PARPi, MEKi and immune checkpoint study underway with Pfizer will be effective with acceptable toxicity in selected patients remains to be determined (NCT03637491).

A number of other alternatives could accrue from a biopsy (or ctDNA) based study. For example, a failure to decrease PARylation could indicate inadequate dosing. Further, since PARPi have been hypothesized to induce synthetic lethality through induction of double stand breaks assessment of the DNA-damage response may provide an indication of efficacy. Indeed, the failure of PARPi to induce a marked and consistent DNA damage response across lesions may be linked to activity. However, a combination of PARP and BRD4 inhibitors might benefit these patients, as we previously showed that BRD4 inhibitors increase DNA damage induced by PARPi through the induction of HRD [28].

*In vitro* models have proven to be a time saving and cost-effective tool for hypothesis testing in cancer research. However, reproducing *in vitro* results in an *in vivo* situation, particularly in patients, has been difficult. Here we were able to demonstrate that the adaptive responses of ovarian cancer cell lines to talazoparib were overall similar to those observed in ovarian cancer patients. As for the patient samples, each cell line has its own genetic and phenotypic characteristics that are translated into changes in specific pathways as analyzed through proteomics. Having a better understanding of these pathways and their impact on drug sensitivity is essential to deliver on the promise of targeted therapies and, in particular, combination therapy. In the case of PARPi treatment, cell lines appear to provide a useful tool to characterize adaptive response and to assess effects of combination therapies. A pitfall that might be encountered is in the choice of technologies used to monitor adaptive responses. In the case of RPPA, only a few hundred proteins can be analyzed, which limits analysis to a restricted number of pathways. To achieve a better characterization of the adaptive response, it might be necessary to use antibodies targeting a broader variety of druggable pathways or alternatively, use other proteomics technologies such as mass spectrometry or impute protein and pathway function through RNA analysis. The FDA already approved drugs targeting a broad range of pathways and several of these pathways were not covered by our RPPA assay. Monitoring the expression of major enzymes involved in those pathways would increase the opportunity to target the right pathways for the right patient.

Protein and genomic biomarkers are increasingly able to predict patient outcome and help identify effective therapeutic options. The use of biomarker driven therapy has improved outcomes for a subset of cancer patients and drives the development of new targeted therapies. However, both the positive and negative predictive value of the biomarkers has been suboptimal. This is potentially due to analysis of a single static biopsy that may have limited information content. Identifying and targeting dynamic changes triggered by targeted therapies in cancer cells provides a potential opportunity to improve patient outcomes. Indeed, the adaptive responses of different lesions to PARPi therapy showed much less heterogeneity than static proteomic patients. A study published by Litton and colleagues demonstrated that four weeks of PARPi as neoadjuvant therapy in *BRCA* mutant breast cancer was sufficient to decrease tumor size in 11 out of 13 patients [65], indicating rapid response after initiation of PARPi treatment. Here, our results indicated that one week of treatment might be sufficient to induce adaptive responses, although impact on tumor growth was unclear. A larger patient cohort will be necessary to determine the ideal treatment duration necessary to detect and analyze adaptive response of the tumor and define the spectrum of adaptive responses that occur during PARPi treatment.

Overall, our study provides a proof of concept that window of opportunity trials can be achieved in ovarian cancer patients. This opens up a valuable opportunity to test new drugs or develop new approaches to treat ovarian cancer patients who have not been treated by multiple other drugs. Moreover, the possibility to assess intratumoral as well as interlesional heterogeneity early during the course of treatment might lead to a better understanding of how ovarian cancer cells adapt to therapy and become resistant, leading to effective combination treatment options. In addition, due to the clear target engagement that was demonstrated after a short duration of treatment, we are now accruing to a neoadjuvant trial of PARP inhibition in the treatment of upfront *BRCA* mutant ovarian cancer. These studies will be necessary to determine whether targeting adaptive responses to PARPi will improve patient outcomes.

## MATERIALS AND METHODS

This was a single arm, open label, window of opportunity study of talazoparib in patients with newly diagnosed advanced stage ovarian cancer. The study (NCT02316834) was conducted by the University of Texas MD Anderson Cancer Center (MDACC) and was supported by the MDACC Ovarian Cancer Moon Shot and Pfizer.

### Patient population

Eligible patients had presumed advanced stage HGS ovarian, fallopian tube, or primary peritoneal cancer. Patients had to be a candidate for primary cytoreductive surgery with ECOG performance status of 0 or 1 and be able to tolerate oral medication. Patients were not permitted to have received any prior cancer therapy. All patients were required to have adequate bone marrow, liver, and renal function. Patients with significant symptom burden including large volume ascites, shortness of breath on exertion, or pain requiring narcotic medication were excluded. All subjects provided written informed consent and the study was approved by the Institutional Review Board.

### Treatment plan

At our institution, patients with presumed advanced-stage ovarian cancer are considered for laparoscopic tumor assessment to determine likelihood of tumor resection to no gross residual disease [66]. Patients scored as < 8 using a validated scoring system proceed to primary cytoreductive surgery at a later date, while patients scored as ≥ 8 are treated with neoadjuvant chemotherapy. For this study, patients were pre-enrolled and consented at the time of preoperative visit for laparoscopic scoring surgery. If the patient received a score of < 8, she went on to receive talazoparib for at least 7 days prior to planned tumor reductive surgery. Talazoparib was administered at a dose of 1 mg orally once daily. Patients received talazoparib until the day prior to surgery or patient withdrawal from study. After recovery from surgery, patients received platinum and taxane cytotoxic chemotherapy as per their primary treating physician.

### Assessments

Toxicities were monitored through the duration of the study and 30 days after cessation of study treatment. Common Terminology Criteria for Adverse Events (CTCAE), Version 4.0 was utilized to grade adverse events.

### Specimen collection

Tissue specimens were collected from four matched sites at the time of laparoscopy and cytoreductive surgery (Supplementary Table 1). Sites included, but were not limited to: ovary, peritoneum, omentum, and diaphragm. Blood was obtained at laparoscopy, tumor reductive surgery and at a 30-day post-surgery visit.

### Reverse phase protein array (RPPA)

Protein extracted from tumor samples and cell lines were analyzed by RPPA as previously described [35, 36, 67]. To emphasize the response of cancer and immune cells to PARPi, we included proteins and particularly phosphoproteins that are involved in major signaling pathways, immune activation and DNA damage response. To improve the signal to noise, we removed structural proteins that are primarily stromal. Data were normalized and heat-maps were generated from the ratio of treated to untreated samples, using unsupervised hierarchical clustering analysis of the samples and Rank-Sum ordering of the proteins. The heat maps were produced using publicly available Cluster 3.0 and TreeView software.

### Cells and reagents

All cell lines were acquired through the MDACC Characterized Cell Line Core and were authenticated by fingerprinting using short tandem repeat testing. The absence of mycoplasma contamination was also verified. All cell lines were maintained in RPMI-1640 supplemented with 5% FBS. Viability assays were performed using Prestoblue cell viability reagent (Thermos Fisher Scientific) according to the manufacturer’s recommendation. Briefly, cells were seeded into a 96-well plate 24 hours before treatment with or without talazoparib, AZD1775 (Wee1 inhibitor) and AZD6738 (ATR inhibitor). Viability was assessed six days post-treatment. For matrigel (3D) experiments, cells were incubated for four days on a thin coat of growth factor reduced matrigel (BD). Cells were then treated with or without talazoparib and AZD6738 for seven additional days. For 3D regrowth experiment, cells were treated for 7 days and were then washed twice before addition of fresh complete media. Viability was assessed seven days after the removal of drugs.

### Pathway analysis

All pathway predictors have been previously described [41], except for the DNA damage and G2/M DNA damage checkpoint predictors that we predefined based on a literature review. Proteins used as predictors of the different pathways are listed in Supplementary Table 2. To determine a pathway score, the protein Z-scores were calculated and all positively associated predictors were summed minus the predictors that are negatively associated with the pathway.

### Statistical analysis

Chou-Talalay combination index (CI) method was used to assess synergism between PARP and DNA damage checkpoint inhibitors. A CI of 1 represents additivity, CI <1 indicates synergism and CI >1 indicates antagonism. A CI <0.5 indicates strong synergism.

## SUPPLEMENTARY MATERIALS


