# Supplementary material for: Adaptive responses in a PARP inhibitor window of opportunity trial illustrate limited functional interlesional heterogeneity and potential combination therapy options
Source: Oncotarget. 2019 May 28;10(37):3533–46. doi: 10.18632/oncotarget.26947 (PMC6544405; doi:10.18632/oncotarget.26947)
Supplement: Supplementary file 1 [file oncotarget-10-3533-s001.pdf]

Adaptive responses in a PARP inhibitor window of opportunity trial illustrate limited functional interlesional heterogeneity and potential combination therapy options

SUPPLEMENTARY MATERIALS

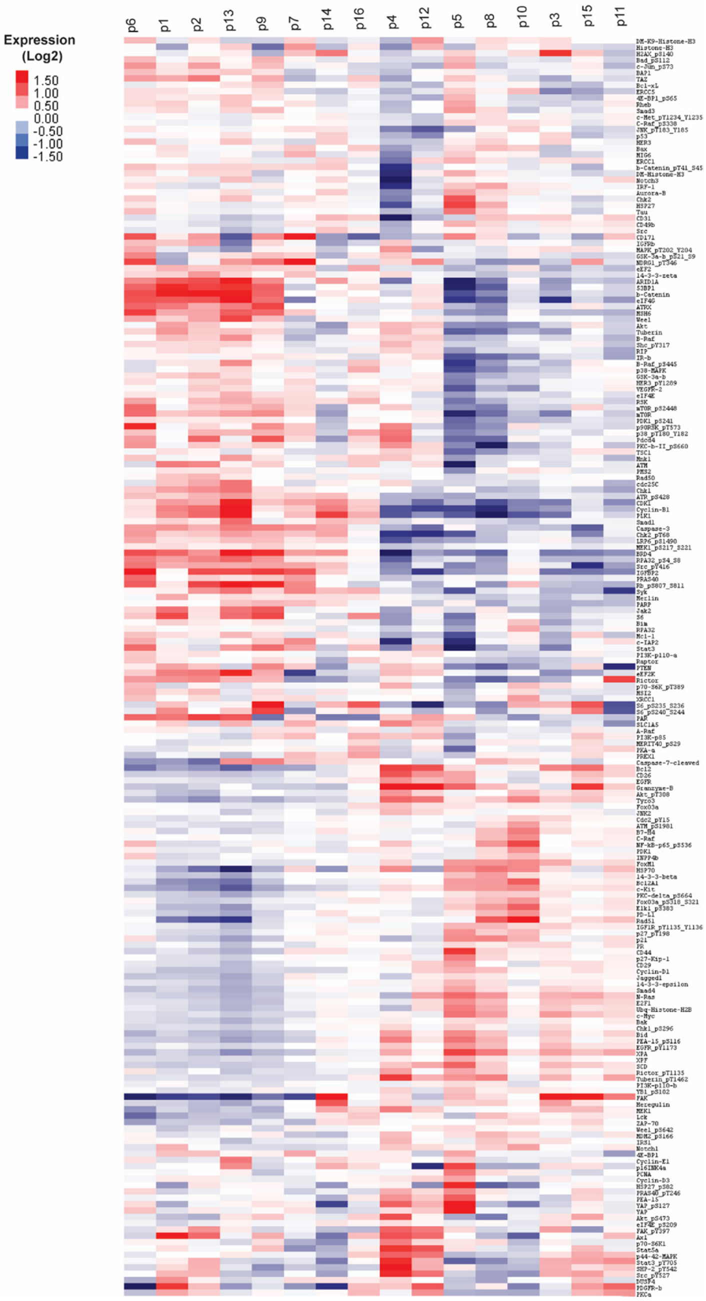

Supplementary Figure 1: HGSOC response to PARP inhibitor. Heat map representing the unsupervised clustering of protein samples analyzed by RPPA. Red and blue colors represent higher and lower expression, respectively. Samples P1-P5 are from patient 1, P6-P10 from patient 2 and P11-P16 from patient 3.

Supplementary Table 1: Tissue of origin of the tumor samples that were collected pre and post talazoparib treatment

Patient	Talazoparib	Samples	Tissue
1	Pre	P1	L Paracolic Gutter Bx
		P2	Pelvic -bx
	Post	P3	L Paracolic Gutter Bx #1
		P4	L Paracolic Gutter Bx #2
		P5	Pelvic-bx
2	Pre	P6	left diaphragm
		P7	omentum
	Post	P8	left diaphragm
		P9	omentum
		P10	peritoneal
3	Pre	P11	pelvic peritoneal
		P12	omentum
		P13	left upper quadrant peritoneum
	Post	P14	pelvc peritoneal
		P15	omentum
		P16	left upper quadrant peritoneum

Supplementary Table 2: Proteins used to predict pathways activity

Pathway	Predictor	Direction
**DNA Damage**	ATM_pS1981	+
	ATR_pS428	+
	H2AX_pS140	+
	RPA32_pS4_S8	+
**G2 Checkpoint**	Cdc2_pY15	+
	Chk1_pS296	+
	Wee1_pS642	+
**Apoptosis**	Caspase-7 (cleaved)	+
	Caspase-3 (active)	+
**Immune Checkpoint**	B7-H4	+
	PD-L1	+
	ZAP-70	+
	Lck	+
**PI3K-Akt**	AKT_pS473	+
	AKT_pT308	+
	GSK3a-b_pS21_S9	+
	P27_pT198	+
	PRAS40_pT246	+
	TUBERIN_pT1462	+
	INPP4B	-
	PTEN	-
**RAS-MAPK**	CJUN_pS73	+
	CRAF_pS338	+
	JNK_pT183_Y185	+
	MAPK_pT202_Y204	+
	MEK1_pS217_S221	+
	P38_pT180_Y182	+
	YB1_S102	+
**Tsc-mTOR**	4EBP1_S65	+
	P70S6K_pT389	+
	MTOR_pS2448	+
	S6_pS235_S236	+
	S6_pS240_S244	+
	RB_pS807_S811	+
**RTK**	EGFR_pY1173	+
	HER2_pY1248	+
	HER3_pY1298	+
	C-MET_pY1234_Y1235	+
	IGFR_pY1135_Y1136	+
	IRS1	+
	SHC_Y317	+
	SRC_Y416	+
	SRC_Y527	+

Supplementary Table 3: Pathway scores measured in patient pre and post treatment samples

	Sample	DNA Damage	G2 Checkpoint	Apoptosis	Immune	PI3K_ AKT	RAS_ MAPK	RTK	TSC_ mTOR	Bcl-2	PDGFR-b	FAK	p16INK4a	S6_pS235_ S236
Pre-Tx	**P1**	0.061	–0.562	–0.048	–0.286	–0.092	–0.012	0.027	0.016	–0.769	0.930	–1.031	0.022	0.429
	**P2**	0.104	–0.364	–0.283	–0.229	–0.061	0.030	0.009	0.100	–0.943	0.293	–0.974	–0.002	–0.082
	**P6**	0.095	–0.295	–0.103	–0.307	0.072	0.108	0.030	0.178	–1.238	–3.377	–1.277	–0.244	–0.283
	**P7**	0.115	0.044	0.452	–0.122	–0.008	0.024	–0.006	0.023	–0.796	–0.483	–0.985	–0.181	–0.324
	**P11**	–0.127	0.046	–0.014	0.056	–0.028	–0.036	0.000	–0.100	0.392	0.685	0.690	–0.435	–1.299
	**P12**	–0.128	0.073	–0.503	–0.050	0.019	–0.227	–0.007	–0.166	1.559	0.750	–0.321	–2.400	–1.728
	**P13**	0.156	–0.146	0.700	–0.350	–0.030	–0.040	0.005	0.124	–1.443	–0.464	–1.237	1.266	0.082
Post-Tx	**P3**	0.162	0.342	–0.183	0.109	0.031	0.033	0.061	–0.035	1.080	–0.083	1.519	0.368	0.530
	**P4**	–0.206	0.073	–0.204	–0.037	0.166	–0.012	0.075	0.031	1.719	0.236	0.164	0.002	–0.609
	**P5**	–0.130	0.326	0.007	–0.064	0.066	0.026	–0.012	–0.082	0.341	–0.237	0.361	2.077	–0.582
	**P8**	–0.061	0.181	0.088	0.284	0.028	–0.018	0.046	–0.132	–0.090	0.083	0.166	–0.922	–0.905
	**P9**	–0.034	–0.361	0.731	–0.191	0.029	0.097	–0.020	0.202	–0.820	–0.119	–0.754	–0.432	2.103
	**P10**	0.156	0.199	0.029	0.406	0.059	0.056	–0.004	0.023	0.093	–0.557	–0.007	–0.941	0.576
	**P14**	0.206	0.058	0.349	0.144	–0.057	–0.009	0.016	–0.051	–0.267	–0.925	1.902	0.860	0.523
	**P15**	–0.016	0.139	–0.570	0.100	0.035	–0.043	0.001	–0.001	1.296	0.432	1.271	0.333	1.268
	**P16**	0.017	0.033	0.041	0.125	–0.002	–0.008	–0.016	0.011	0.090	0.277	0.007	0.374	1.246

Supplementary Table 4: Pathway scores measured in untreated and PARPi treated cell lines

	Cell line	BCL2	G2-M Checkpoint	DNA Damage	Apoptosis	RTK	RAS-MAPK	Tsc-mTOR	PI3K-Akt	P16INK4a	PDGFRb	FAK	S6 pS235/ S236
CTRL	**OVCAR5**	–0.151	–0.088	0.032	0.348	0.047	–0.010	–0.027	–0.109	–0.997	–0.115	0.009	–0.693
	**A2780CP**	0.037	0.011	0.257	0.462	–0.014	–0.097	–0.548	0.341	–0.872	5.705	0.625	–2.903
	**IGROV1**	–0.301	0.014	0.022	0.149	0.060	0.145	–0.107	0.669	–0.001	–0.174	–0.344	0.230
	**OAW42**	–0.137	0.074	0.117	0.135	0.012	0.018	–0.255	0.281	0.034	0.665	–0.054	–1.569
	**CaOV3**	–0.205	–0.122	–0.061	0.131	0.012	0.170	0.066	–0.129	1.091	–0.450	–0.107	1.407
	**HEYA8**	–0.190	–0.091	0.252	0.216	0.031	0.248	–0.717	–0.108	–1.140	–0.233	0.516	–3.451
	**OVCAR8**	–0.155	0.193	0.088	0.198	–0.062	–0.008	–0.082	–0.090	1.721	5.503	0.979	–0.298
PARPi	**OVCAR5**	–0.127	–0.043	0.043	0.393	0.020	0.047	0.075	–0.050	–0.747	–0.045	–0.020	–0.160
	**A2780CP**	0.058	0.243	0.105	0.486	–0.063	0.008	0.128	0.378	–0.732	4.177	0.199	–0.437
	**IGROV1**	–0.310	0.020	0.033	0.136	–0.018	0.164	0.039	0.696	0.001	0.141	–0.395	0.605
	**OAW42**	–0.037	0.142	0.088	0.140	–0.053	0.081	0.287	0.329	0.122	0.215	–0.047	0.866
	**CaOV3**	–0.174	–0.104	–0.002	0.204	–0.034	0.286	0.246	0.013	1.122	–0.461	–0.128	2.193
	**HEYA8**	–0.171	–0.129	0.258	0.200	0.018	0.270	–0.325	–0.110	–0.787	–0.169	0.328	–2.368
	**OVCAR8**	–0.166	0.343	0.134	0.402	–0.129	0.256	0.337	0.033	1.645	4.662	0.709	1.693
